# Simple visual stimuli are sufficient to drive responses in action observation and execution neurons in macaque ventral premotor cortex

**DOI:** 10.1371/journal.pbio.3002358

**Published:** 2024-05-20

**Authors:** Sofie De Schrijver, Thomas Decramer, Peter Janssen

**Affiliations:** 1 Laboratory for Neuro- and Psychophysiology, Department of Neurosciences, KU Leuven and the Leuven Brain Institute, Leuven, Belgium; 2 Research group Experimental Neurosurgery and Neuroanatomy, KU Leuven and the Leuven Brain Institute, Leuven, Belgium; Oxford University, UNITED KINGDOM

## Abstract

Neurons responding during action execution and action observation were discovered in the ventral premotor cortex 3 decades ago. However, the visual features that drive the responses of action observation/execution neurons (AOENs) have not been revealed at present. We investigated the neural responses of AOENs in ventral premotor area F5c of 4 macaques during the observation of action videos and crucial control stimuli. The large majority of AOENs showed highly phasic responses during the action videos, with a preference for the moment that the hand made contact with the object. They also responded to an abstract shape moving towards but not interacting with an object, even when the shape moved on a scrambled background, implying that most AOENs in F5c do not require the perception of causality or a meaningful action. Additionally, the majority of AOENs responded to static frames of the videos. Our findings show that very elementary stimuli, even without a grasping context, are sufficient to drive responses in F5c AOENs.

## Abstract 2

Neuronal activity during action execution and action observation is ubiquitous in the primate brain but it is not clear which are the minimal visual features that drive this activity. This study shows that neurons active during action observation and action execution respond to surprisingly simple visual stimuli which do not contain any meaningful action.

## Introduction

Activity during action execution and action observation is ubiquitous in the primate brain. Over the past 3 decades, this intriguing neuronal characteristic has been described in the macaque ventral [1,2] and dorsal premotor cortex [3–5], primary motor [6–8], supplementary motor [9], dorsal prefrontal [10], posterior parietal [9,11–14], and medial parietal cortex [15]. In parallel, numerous functional magnetic resonance imaging (fMRI) studies (reviewed in [16]) and 1 single-cell study [17] have provided evidence for activity during both action execution and action observation in the human brain. However, no study has established which are the minimal visual features that drive neurons active during action execution and action observation, for which we will use the term action observation/execution neurons (AOENs; as in [17]). To avoid any association with mirror neurons and their putative role in action recognition, we prefer the neutral term AOEN to simply describe all neurons that fired during action execution and action observation. Previous studies have shown responses of single AOENs to moving objects causally interacting with other objects in macaques [18], and fMRI activations during observation of moving objects that overlapped but were distinguishable from activations during action observation in humans [19,20]. Bonini and colleagues showed that a subset of AOENs also responds to a static object in the context of a grasping task [21,22]. Here, we wanted to determine whether AOENs in the F5c subsector of the ventral premotor cortex (PMv) fire during specific epochs of the filmed grasping movement or during the entire grasping action, and to what extent AOENs in F5c also respond to visual stimuli in which no meaningful action, no grasping context, and no perception of causality were present.

Using single-cell recordings with chronically implanted multielectrode arrays, we tested whether meaningless stimuli could also activate AOENs in the F5c sector of the macaque ventral premotor cortex, where AOENs were originally discovered. We found that the large majority of AOENs responded in a specific phase of the action and even when a simple object was moving along the same trajectory as the hand in the absence of a graspable object, indicating that for the majority of AOENs in F5c, meaningful actions are not required for their activation.

## Results

**Fig 1:**
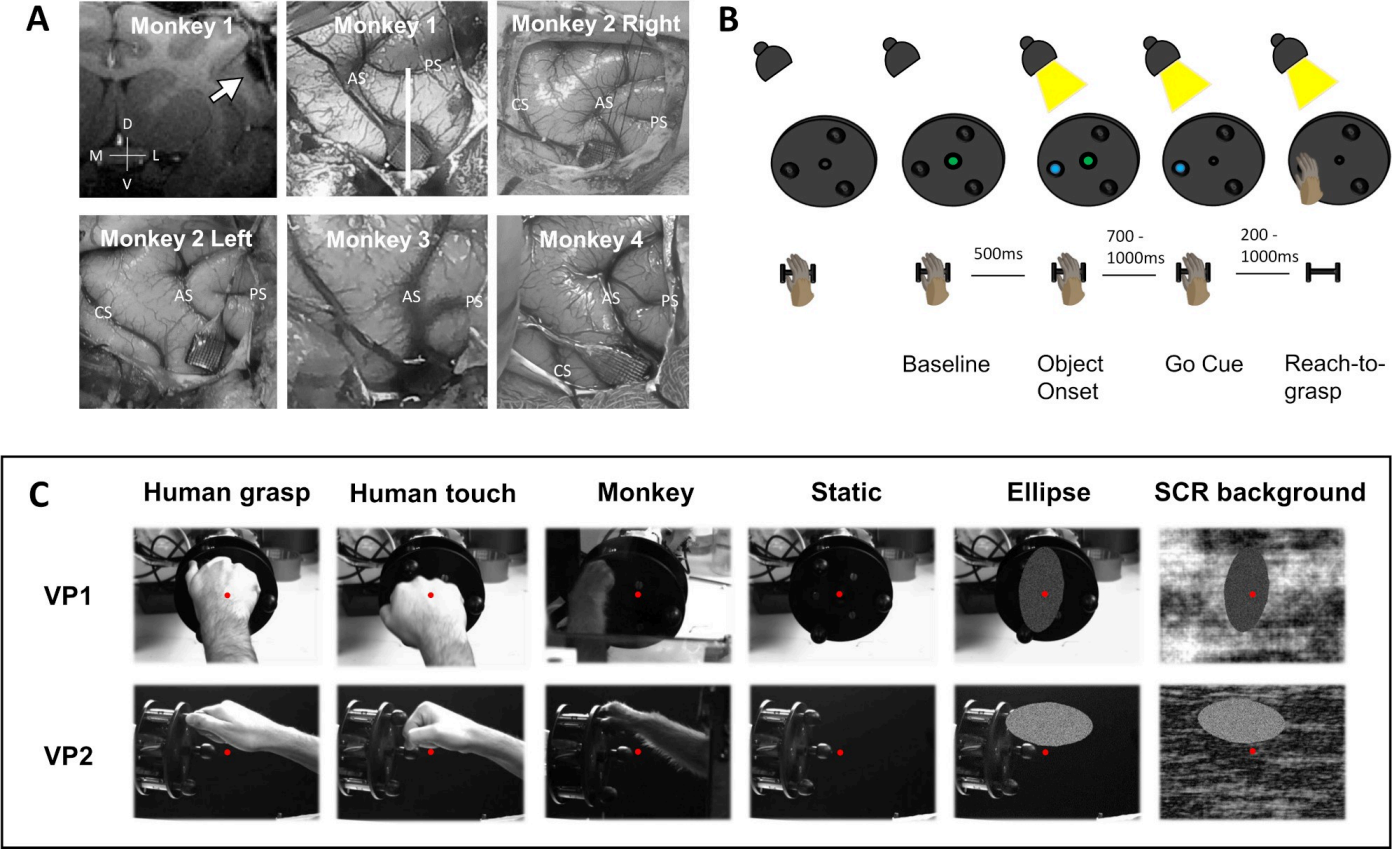
Array implantations and behavioral tasks. (**A**) Locations of implanted Utah arrays in area F5c. Top left: coronal anatomical MRI section of Monkey 1 (white line on implantation picture) with implanted array (white arrow). Axes of the MRI section: M (medial), L (lateral), D (dorsal), and V (ventral). Sulci on the implantation pictures: CS (central sulcus), AS (arcuate sulcus), and PS (principal sulcus). (**B**) Schematic representation of the temporal sequence of the visually guided grasping task, created with BioRender.com. (**C**) Videos shown during the action observation task. Each video was shown from 2 perspectives: point of view of the monkey (Viewpoint 1, VP1) and side view (Viewpoint 2, VP2). The red dot depicts the fixation point.

We recorded the activity of 346 single F5c neurons (SUA) (60% of 586 isolated neurons) and 529 multiunit (MUA) sites (47% of 1,133 recording sites), which were responsive during visually guided grasping, in 15 recording sessions (3 for each implantation). We were able to visualize the multielectrode arrays using anatomical MRI (Fig 1A) with a custom-built receive-only coil (inner diameter 5 cm). Based on these MR images, we observed that the electrode tips were located at a depth of 1 to 2 mm from the surface of the brain (Fig 1A, upper left panel).

### Grasping activity in ventral premotor area F5c

**Fig 2:**
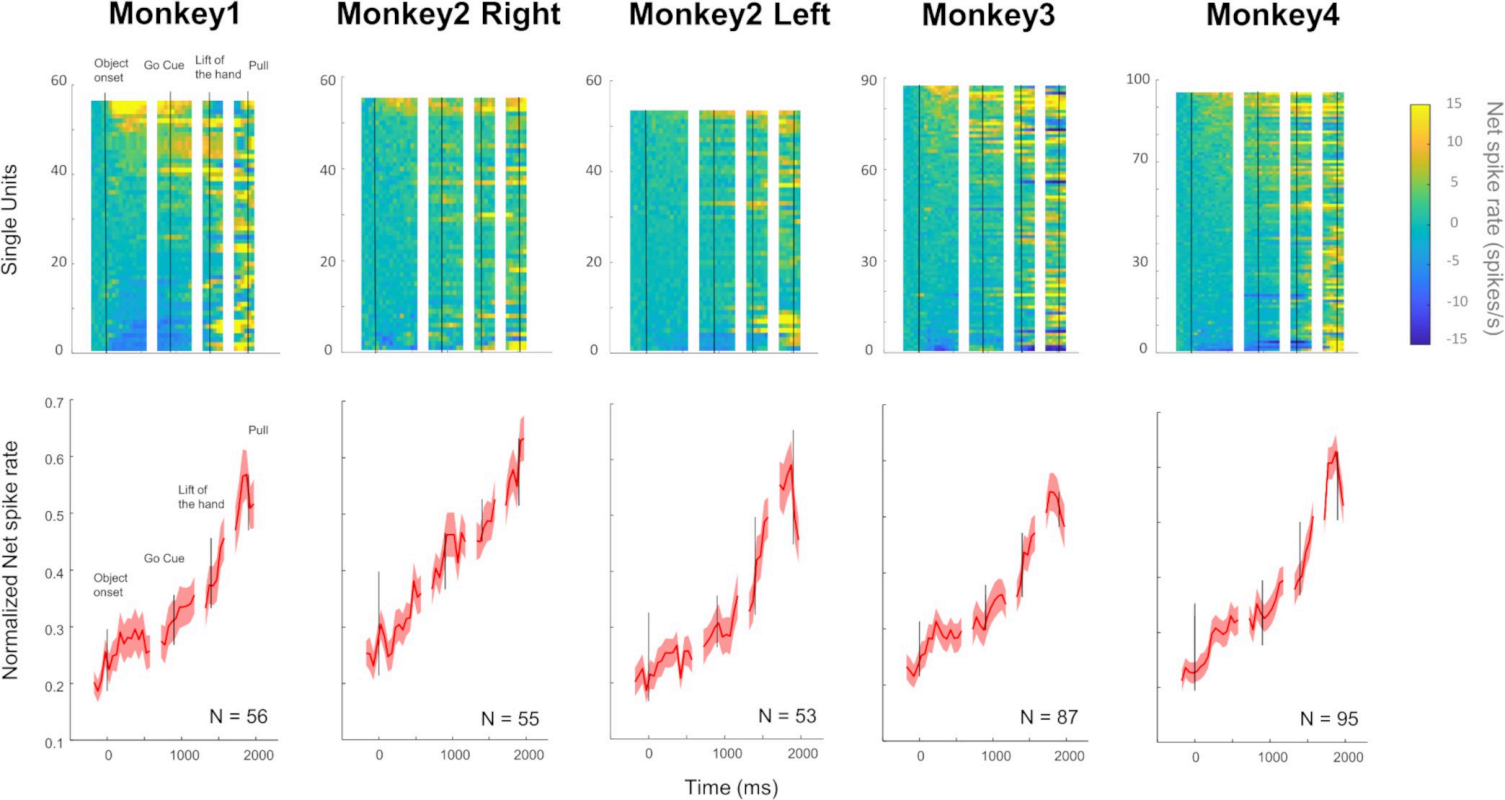
Grasping activity in ventral premotor area F5c. Top: color plots of the net spike rate of each SUA, ranked based on their visual responsiveness during object onset. Bottom: average normalized net spike rate (±SEM) of all SUA of each monkey aligned on the 4 events of the VGG task: Object onset, Go cue, Lift of the hand, and Pull.

Fig 2 shows the average response of F5c neurons during the visually guided delayed grasping task. The graphs show the average normalized (divided by the maximum) task-related activity in 4 epochs of the task: Object onset, Go Cue, Lift of the hand, and Pull of the object. Results were similar between the 5 implantations and were therefore combined for all subsequent analyses. In general, the average SUA remained relatively low in the first 200 ms after object onset, became higher around the Go cue, and rose rapidly after the Lift of the hand until the Pull of the object (Kruskal–Wallis on the average activity of the 5 implantations in the 4 epochs, F [3] = 208.69, *p* = 5.58 × 10^−45^). The steep increase in spiking activity after Lift of the hand was observed in all monkeys when the movement was performed in the light, and most SUA (105/162, 65%) and MUA sites (72%) that were tested in the dark were also responsive during grasping in the dark. Thus, F5c neurons generally showed the highest activity after the hand started moving towards the object. Because the 3 objects were identical, only 22% (77/346) responded significantly differently (Kruskal–Wallis, *p* < 0.05) during grasping of the 3 objects. These response differences during grasping of identical objects were most likely evoked by the different reach trajectories.

Additionally, 161 SUA were negatively modulated during the grasping task. On average, SUA decreased after Object onset in these neurons and decreased even further during the reach-to-grasp movement until the Pull of the object (S1A Fig).

### Responses of F5c neurons during action observation

**Fig 3:**
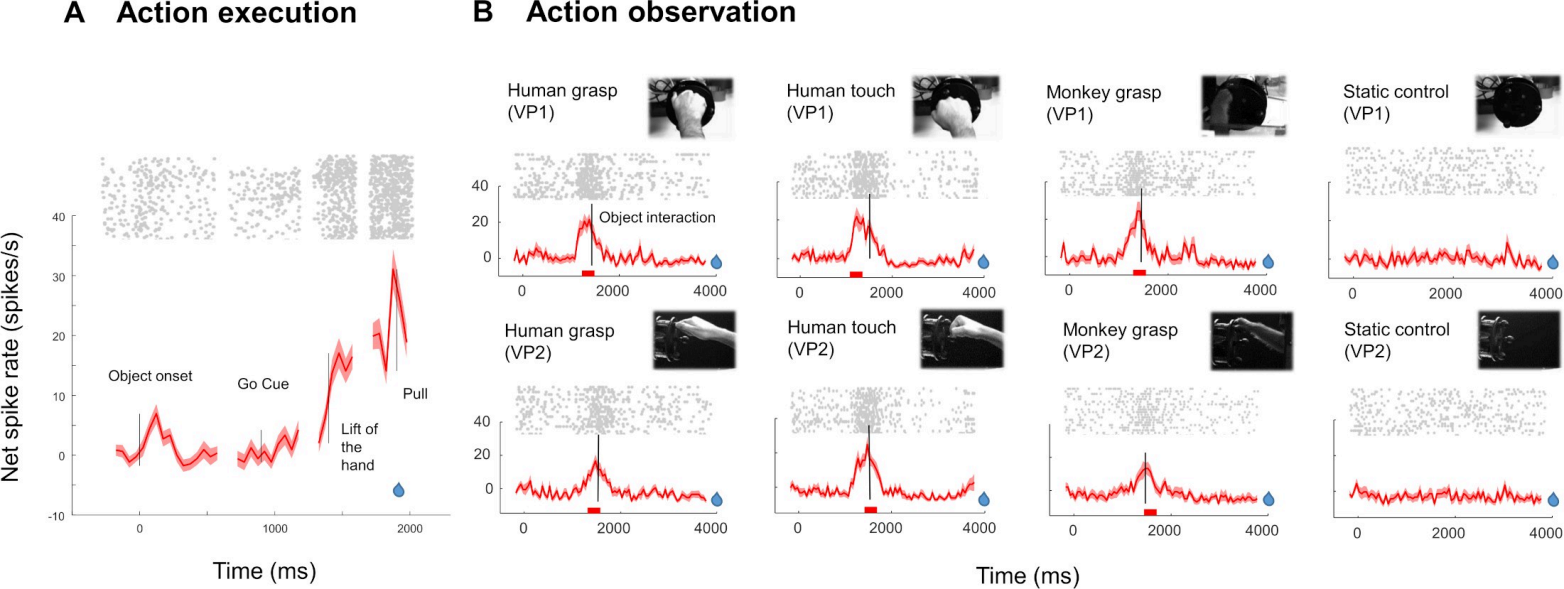
Response of an example F5c neuron during action execution and action observation. (**A**) Average net spike rate (±SEM) of an example neuron during the VGG task. (**B**) Average net spike rate (±SEM) of the same neuron during the action observation task for the 2 viewpoints (VP1 = filmed from the point of view of the monkey, VP2 = filmed from the side). Red bars below the line plots indicate the window of analysis. Blue drops indicate the moment of reward delivery.

The single neuron example in Fig 3 showed strong activation during the grasping task (Action execution), with a weak response to Object onset and maximal activity around the Pull of the object (Fig 3A). This example neuron also responded during passive fixation of a video of a human or a monkey hand performing the same grasping action (Human Grasp and Monkey Grasp, Viewpoint 1 or Viewpoint 2, Fig 3B), and to a video of a human touching the object (Human Touch), but not to a static frame of the video (Static control, Mann–Whitney U test comparing responses to the preferred action video and the static video, z = 9.1463, *p* = 5.89 × 10^−20^). Although clearly responsive to the action videos (spike rate more than 3 standard errors (SEs) above the baseline firing rate), the example neuron did not differentiate well between the 2 viewpoints and the 3 action types. Indeed, a 2-way ANOVA with factors *viewpoint* (Viewpoint 1 and Viewpoint 2) and *action type* (Human Grasp, Human Touch, and Monkey Grasp) revealed no significant main effect of *action type* (F [2] = 0.53, *p* = 0.5875), no significant main effect of *viewpoint* (F [1] = 3.63, *p* = 0.0591) and no significant interaction (F [2] = 0.53, *p* = 0.5911). A second prominent feature illustrated in this neuron is the phasic nature of the action observation responses. In Fig 3B, zero indicates the start of the video and the vertical line the time point at which the hand makes contact with the object. Intriguingly, the example neuron did not respond in the first second after the onset of the video but peaked around the time that the hand made contact with the object (from 290 ms before until 9 ms after object interaction in the different action videos, prominence = 13 to 25 spikes/s for the different action videos; see Methods).

In our population of task-related F5c neurons, 213 SUAs (62%) were also significantly modulated during action observation (AOENs). As in the example neuron, most SUAs (190 neurons, 89%) did not differentiate between the different action types, i.e., AOENs that were broadly congruent (2-way ANOVA with factors *perspective* and *action type*, main effect of *action type* not significant [2]). Likewise, the peak spiking activity of all AOENs was highly correlated between the Human Grasp and the Human Touch videos (r = 0.78, *p* = 1.03 × 10^−44^, S2A Fig), and only 2% of AOENs were selective for one of the 2 types of action (*p* < 0.05 post hoc tests), implying that the specific movements of the fingers were weakly encoded. Furthermore, a small minority of the neurons (4%, *p* < 0.05 post hoc tests) differentiated between a monkey and a human grasping the object in the video (r = 0.82, *p* = 1.78 × 10^−53^, S2B Fig). A subset of 37 F5c AOENs (17%) preferred a specific viewpoint of the video (d’ > 0.4, S2C Fig). More than 65% (25/37) exhibited a preference for Viewpoint 2 in which the action was filmed from the side, which may be related to the better visibility of the action filmed from the side.

**Fig 4:**
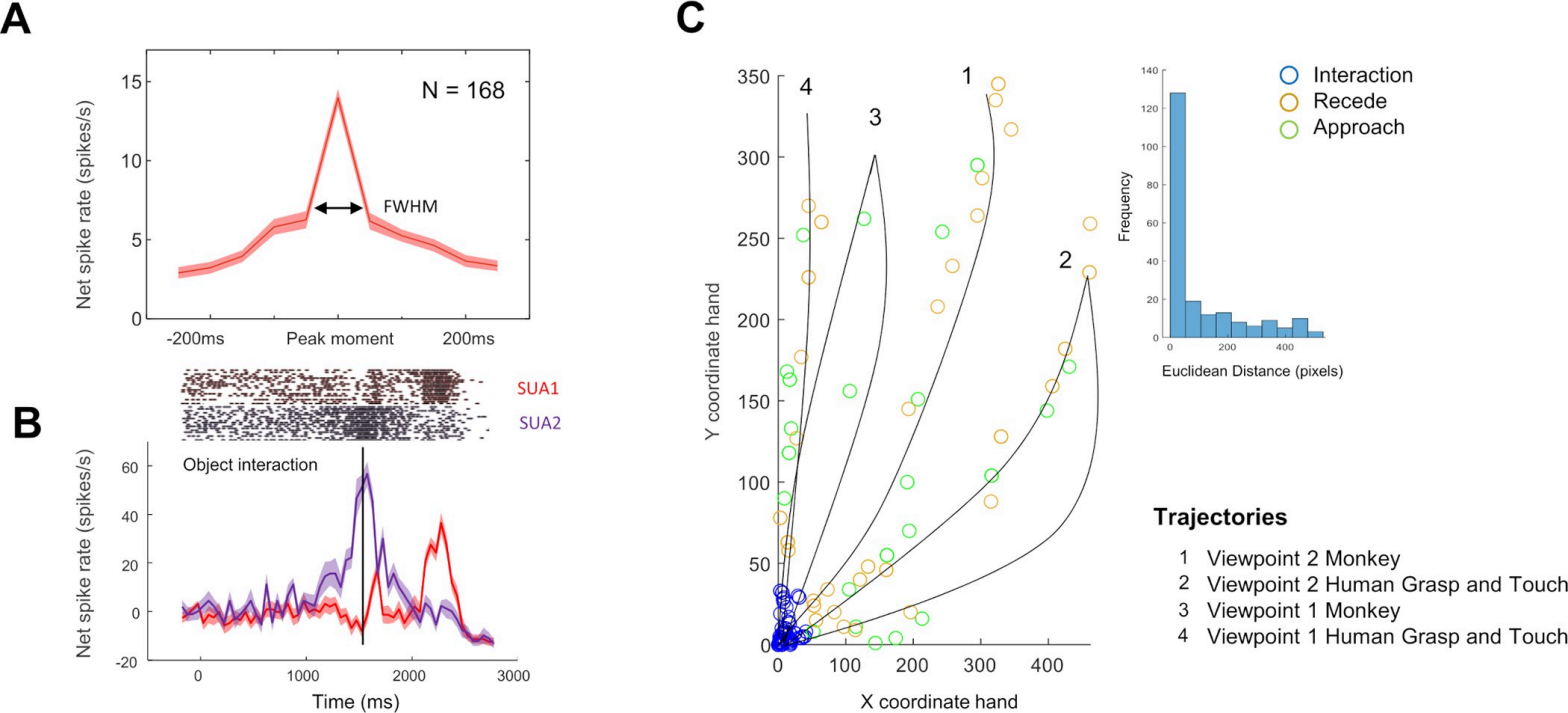
Phasic responses during action observation. (**A**) Average peak response (±SEM) of 168 AOENs (red) plotted in a 500-ms interval around the peak. The arrows indicate the full width at half max (FWHM). (**B**) Average net spike rate (±SEM) of 2 example neurons that were recorded on 1 electrode during the action observation task. Data aligned on video onset with the black line depicting the moment of object interaction. (**C**) Position of the hand relative to the object in the preferred action video at maximal spiking activity. Colors indicate the phase of the movement: green (Approach), blue (Object interaction), and ocher (Recede). Black lines represent an approximation of the trajectory of the hand in the different action videos. Histogram in the inset shows the Euclidean distances between the hand and the object at maximal spiking activity.

The example neuron in Fig 3B did not fire during the entire action video, but only in a specific 750 ms long epoch, with a maximum immediately before the hand made contact with the object. We identified peaks in the firing rate during action observation using the Matlab function findpeaks on the responses to the preferred action video (i.e., the video eliciting the highest peak firing rate). Based on our criterion (see Methods), the large majority of SUA (*N* = 168, 79%) sites were tuned to a specific epoch of the action videos. For those tuned neurons, we then identified the time bin with the highest spike rate during the preferred video for each neuron, plotted the average net spike rate (i.e., baseline activity subtracted) in an interval of 500 ms around the peak firing rate and calculated the full width at half maximum (FWHM) around the peak activity to characterize the degree of tuning (Fig 4A). The average activity of AOENs during action observation showed a clear peak during the action video, with a FWHM equal to 85 ms (Fig 4A). These results illustrate that most AOENs in our sample did not discharge during the entire duration of the video but rather in a narrow interval during a specific epoch of the video. Because these highly phasic responses sometimes occurred both in the approach and in the recede phase of the video and frequently occurred in a short period in each interval, only 31% of the neurons were significantly selective for one of the 3 intervals (approach, object interaction, and recede; Kruskal–Wallis, *p* < 0.05).

One could argue that the phasic responses were induced by a single aspecific factor such as muscle contractions, attention, or reward expectation during the action video. However, such an aspecific factor should have an effect on all neurons that were recorded simultaneously. Fig 4B shows 2 neurons that were recorded simultaneously on 1 electrode and that discharged at different moments in time. If the phasic responses would have been induced by covert hand movements of the monkey or by attention, these neurons would have had a highly similar response pattern. Additionally, we observed a high trial-by-trial reliability (raster plots of Fig 4B). Furthermore, in every recording session, we observed multiple neurons that fired maximally at different moments in the action video (S3A Fig). Moreover, we analyzed the electromyographic (EMG) recordings of the most important hand and arm muscles during an additional recording session in Monkey 3. We found no significant correlation between the average firing rates of the 12 recorded AOENs and the rectified EMG signal (Pearson correlation coefficients, median = 0.11, all *p* > 0.05, S3B Fig), indicating that the phasic responses of F5c AOENs were not induced by covert hand or arm movements during the action video. Finally, the phasic responses occurred at different time points in the video, making it highly unlikely that the neurons were modulated by reward expectation since the reward was always given 4 s after the start of each video.

Next, we wanted to investigate the relation between the time at which the neural activity peaked and the phase of the action in the video. To that end, we determined the coordinates of the hand 50 ms before the peak firing rate (to account for the neural latency) and calculated the Euclidean distance between the hand and the object at that moment. Fig 4C illustrates these locations of the hand relative to the object (at the origin in the graph) at the maximum firing rate. The majority of the neurons (60%, blue circles) fired maximally around hand–object interaction, when the hand was within a 50-pixel radius (corresponding to 1.9 visual degrees) around the object. The example neuron in Fig 3 illustrates this predominant response pattern with a steep increase in activity immediately before the hand interacted with the object. The remaining neurons responded maximally at different moments in time, either when the hand was approaching the object (14%, green circles) or when the hand was receding (26%, red circles, black lines illustrate an approximation of the hand trajectories in Fig 4C). The distributions of the Euclidean distance between the hand and the object at the peak firing rate were highly positively skewed (inset in Fig 4C, Shapiro–Wilk test, *p* = 7.7716 × 10^−16^). Thus, the majority of AOENs in area F5c discharge when the hand interacts with the object and they faithfully represent the location of the hand with respect to the object for a specific viewpoint. Indeed, the distance of the hand to the object at the peak firing rate did not correlate between the 2 viewpoints (r = 0.12, *p* = 0.12).

**Fig 5:**
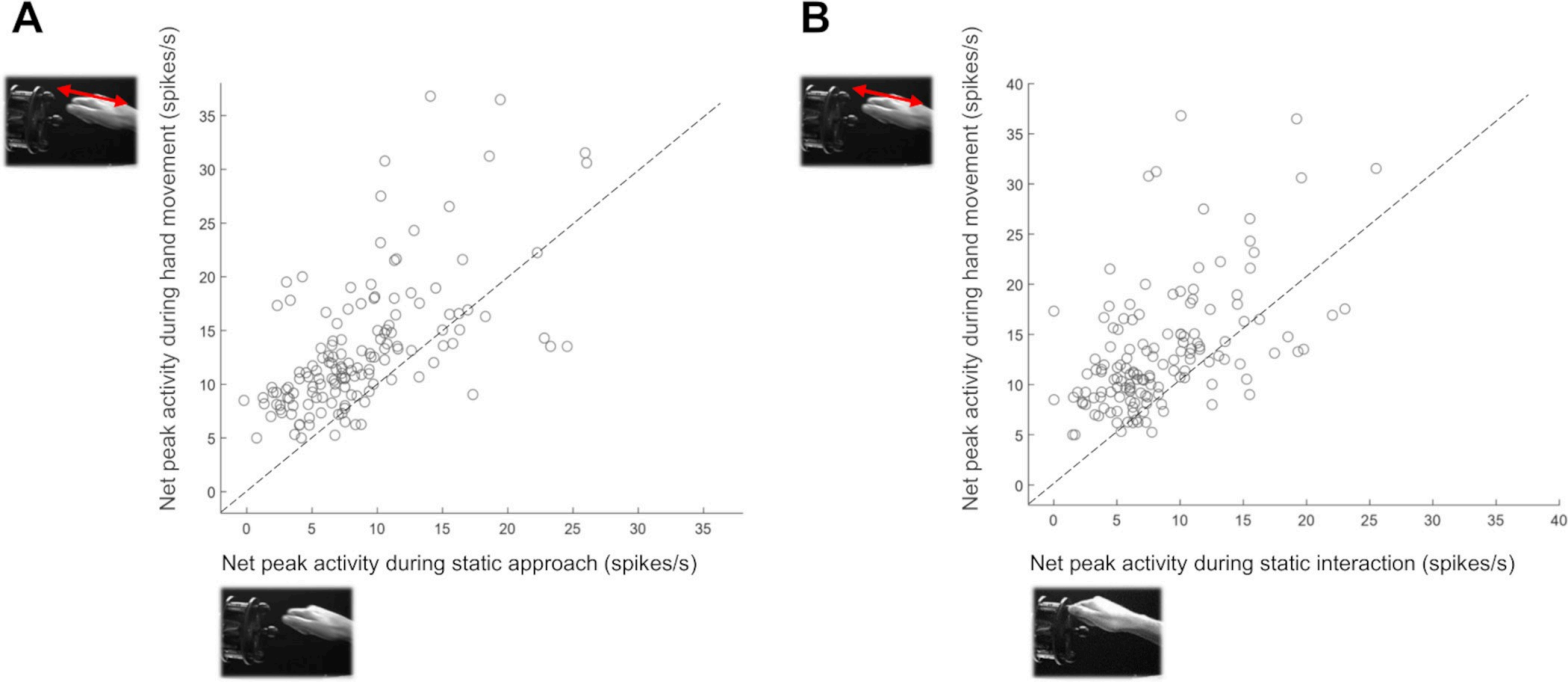
Neural responses to static frame videos. (**A**) Net peak activity during the movement epoch of the action video (red arrow indicates the grasping movement of the hand) compared to the net peak activity during the Static Approach video (i.e., video of a static frame wherein the hand is halfway the reach trajectory). (**B**) Same as (**A**), but comparing the net peak activity during the action video to the net peak activity during the Static Interaction video. The dashed line represents the equality line.

To test the possibility that AOENs merely responded to a static frame of the action videos, we also presented static images in which either the hand was approaching the object (Static Approach) or interacting with the object (Static Interaction) in a subset of neurons (*N* = 144 AOENs). For each AOEN, the viewpoint of the static frame video was matched to the viewpoint of the preferred action video. We then compared the peak firing rate of the action video with the peak firing rate during presentation of the static frames. Although the average peak firing rate to the static frame was lower compared to that in the action videos (8.6 spikes/s compared to 13.2 spikes/s), the correlations between the action video responses and the static frame responses were high (r = 0.61, *p* = 3.9858 × 10^−16^ for the Static Approach video and r = 0.52, *p* = 1.9114 × 10^−11^ for the Static Interaction video) (Fig 5). These results suggest that the static frames of the action videos could partially account for the observed phasic AOEN responses.

### Visual selectivity of AOENs

**Fig 6:**
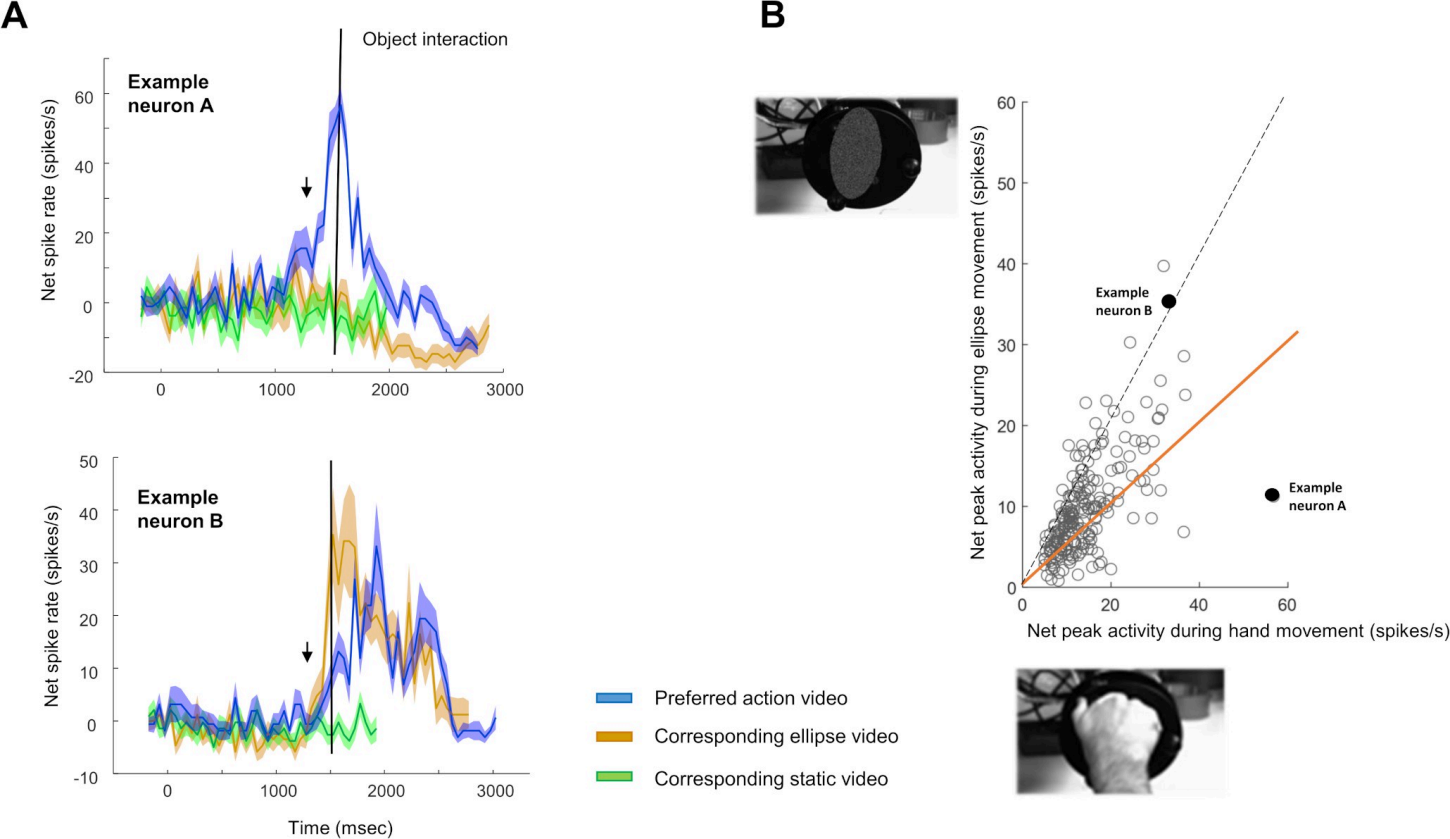
AOEN responses to an abstract moving shape. (**A**) Average net spike rate (±SEM) of 2 example neurons during 3 videos: the preferred action video (blue), the corresponding ellipse video (ocher), and the corresponding static control video (green). The black line indicates the moment of object interaction in the action video. The arrow indicates the moment of object interaction in the ellipse video. (**B**) Maximal spiking activity during the preferred action video plotted against the maximal spiking activity during the corresponding ellipse video. The orange line represents the 50% criterion to define ellipse neurons.

The crucial test in our experiment was the comparison between the action video responses and the responses to a simple shape moving towards the object along the same trajectory as the hand in the action video, but without a meaningful action and without any percept of causality. Fig 6A shows the responses of 2 AOENs to the preferred action video (blue, aligned to the onset of the video), the corresponding ellipse control video (ocher) and the static control (green). The first example neuron (Fig 6A, top) responded strongly to the action video (Human Grasp) with a sharp peak at the time of hand–object interaction (FWHM = 215 ms) but did not respond to the ellipse video or to the static control. However, the second example neuron (Fig 6A, bottom) responded to both the action video and the ellipse video, with a maximal firing rate that was as high for the ellipse video as for the action video, although slightly earlier in time, while the static control did not elicit any response. Thus, the activity pattern of this second example neuron implies that a simple shape moving in the visual field was sufficient to elicit a robust neural response. Note that, similar to the neural responses during the action videos, a considerable number of AOENs (125/213 or 59%) showed a highly phasic response during the ellipse video, with an average FWHM of 74.

Remarkably, 74% (158/213) of the F5c AOENs responded during the ellipse video with a peak discharge rate that was at least 50% of the response to the action video. We refer to these neurons as “ellipse” neurons. Fig 6B illustrates that a large fraction of AOENs (48%) even responded to the ellipse video at more than 70% of the action video response. The action video responses correlated strongly with the corresponding ellipse video responses across the population of AOENs (r = 0.66, *p* = 9.99 × 10^−28^). The majority of ellipse neurons (77%) was tuned to a specific epoch of the action video (findpeaks, prominence > 0.8), similar to non-ellipse neurons (92%). When restricting the analysis to AOENs that were significantly modulated during grasping in the dark (*N* = 105), we observed similar response properties, i.e., 73% were ellipse neurons and the correlation between the action video responses and the ellipse video responses was remarkably high (r = 0.73, *p* = 8.98 × 10^−19^). Thus, most F5c neurons that respond during action execution and action observation also respond to a simple shape moving towards an object. Analogous to the action video responses, the majority of ellipse neurons (60%) peaked around the time the ellipse was near the object, whereas a minority of these neurons fired maximally in the approach (13%) or recede (27%) phase. Note that only 10% of the AOENs showed a significant positive correlation between the action video and the static video responses, and, similarly, only 11% of the AOENs showed such a positive correlation between the ellipse video and the static video responses.

**Fig 7:**
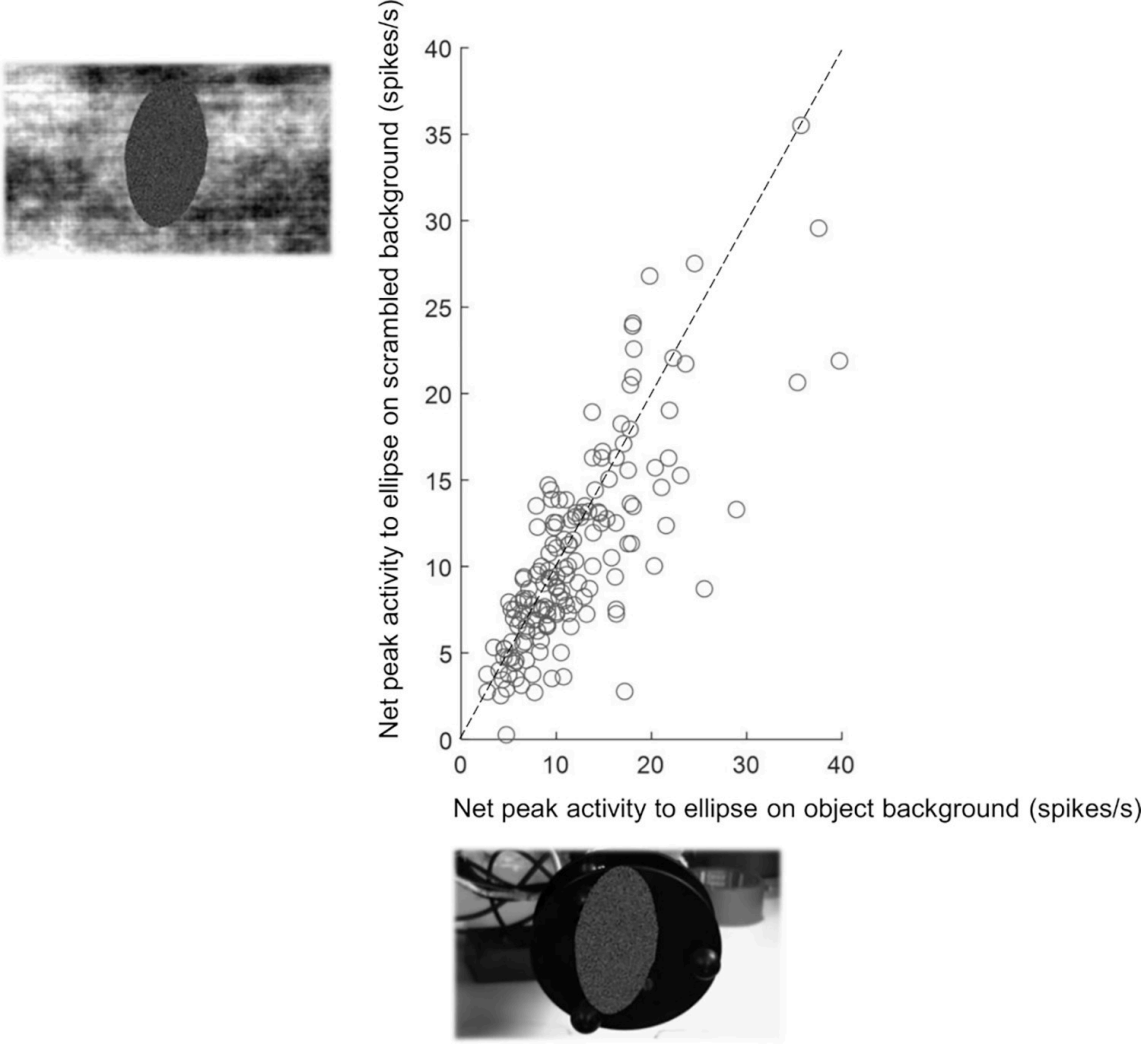
Ellipse neuron responses to a moving shape on a scrambled background. Net peak activity during the ellipse video (perspective of the preferred action video) plotted against the net peak activity during the corresponding scrambled background video for all ellipse AOENs.

Because the videos of the ellipse moving towards an object might induce the impression of causality, we tested to what extent the presence of the object was necessary for the F5c AOENs in our sample. Therefore, we also presented videos of the ellipse moving along the same trajectory on a scrambled background in which no object was visible, in interleaved trials. Even more surprisingly, an ellipse moving on a scrambled background was highly effective in driving AOEN responses. Indeed, the correlation between the responses to the 2 control videos was very high (r = 0.80, *p* = 4.18 × 10^−48^). When restricting the analysis to ellipse neurons (i.e., neurons for which the ellipse response reached at least 50% of the action video response), this correlation was equally high (r = 0.78, *p* = 7.64 × 10^−34^, Fig 7). Only a small minority of F5c ellipse neurons (8% or 13/158) could significantly differentiate between the normal and the scrambled background videos (Mann–Whitney U test, *p* < 0.05). Thus, F5c neurons responding to action execution and action observation generally respond well to very abstract dynamic stimuli such as a simple shape moving in the visual field in the absence of an object.

Furthermore, we investigated whether the ellipse neurons were simply selective for direction of motion by comparing the spiking activity when the ellipse moved on a scrambled background either towards or away from the position of the object in the ellipse video with intact background. Using the 2 viewpoints of the videos, we could compare 2 possible orientations (vertical and horizontal) and 4 directions (movement upwards, downwards, left, and right). Of the 158 ellipse neurons, only 33 (21%) had a significant preference for 1 orientation of the ellipse (Mann–Whitney U test, *p* < 0.05). A small minority of the F5c ellipse neurons was direction selective (10% for the vertical directions, and 18% for the horizontal directions, Mann–Whitney U test, *p* < 0.05). Taken together, these results imply that for most F5c ellipse neurons, a basic selectivity for the direction of motion could not account for their action observation responses.

### Visual selectivity of neurons inhibited during the VGG task

In our sample of F5c neurons, we observed 161 units that were negatively modulated during the VGG task. More than half (88/161, 55%) also increased their firing rate during the observation of filmed actions. Similar to the AOENs with an excitatory response during the VGG task, the majority (66%) also responded to an ellipse moving on the screen with a peak response that was more than 50% of the peak response to the preferred action video (i.e., ellipse AOENs, S1B Fig). Additionally, we observed a high correlation between the peak responses of these ellipse AOENs to the ellipse video with the object in the background and the ellipse video with a scrambled background (r = 0.83, *p* = 3.86 × 10^−16^, S1C Fig), implying that the majority of these AOENs do not require the presence of an object. A subset of the neurons that were inhibited during the VGG task also showed inhibition during action observation (43%), whereas only 2% was not responsive during action observation.

### Suppression AOENs

As described by Kraskov and colleagues [8], neurons in primary motor cortex that are positively modulated during grasping can also be negatively modulated during action observation (i.e., suppression AOENs). In our population of grasp-responsive F5c neurons, 16% (56/346) showed a significant inhibitory response to the observation of at least 1 action video. Surprisingly, these suppression AOENs also responded strongly to the ellipse videos. S4A Fig shows the activity of 2 suppression AOENs during the preferred action video and the corresponding ellipse video. The activity of example neuron A decreased distinctly in both videos around object interaction and remained low for the rest of the video. In contrast, example neuron B was inhibited in the receding phase of the action video but excitatory in the receding phase of the ellipse video. Overall, we found a high correlation between the minimal spiking activity during the action video and the spiking activity at the same time point in the ellipse video (r = 0.84, *p* = 3.31 × 10^−16^, S4B Fig). These results suggest that suppression AOENs respond to the movement of an abstract shape in a similar way as to the action video.

### Multiunit responses in F5c

We also recorded 529 F5c MUA sites that were significantly modulated during the action execution task (VGG task) with a steep increase in the firing rate during the reach-to-grasp movement (S5A Fig). Overall, the MUA results were highly comparable to our findings in SUA. More than half of the MUA sites (289/529) also responded during the action observation task. Similar to the SUA, we found low selectivity for the type of action (grasping versus touching, r = 0.76, *p* = 1.31 × 10^−55^), the actor (monkey versus human, r = 0.73, *p* = 1.46 × 10^−50^), and the perspective (only 12% preferred a specific viewpoint). Furthermore, the majority of the MUA sites (76%) showed a clear tuning to a specific moment in the action video (FWHM = 98), with a preference for the moment of object interaction (S5B Fig). Similar to the SUA, 72% of the MUA also responded to the observation of the movement of an ellipse (i.e., ellipse sites, S5C Fig). Only few ellipse MUA sites (14/289, 5%) could differentiate between the ellipse movement on the normal background and the scrambled background, implying that the majority of the F5c MUA sites with AOE activity also respond to the movement of an abstract shape in the absence of a graspable object (S5D Fig). In the AOE sites where we could record multiple SUAs, 65% (15/23) contained only AOENs, whereas 35% (8/23) contained a mix of AOENs and non-AOENs.

## Discussion

In a large population of F5c neurons (346 SUA and 529 MUA sites) responsive during object grasping, a subpopulation (62% SUA and 55% of MUA) also responded to videos of grasping actions (AOENs). We found that the activity of the majority of F5c AOENs sharply rose during a specific epoch of the observed grasping action, primarily around the time that the hand made contact with the object, although smaller numbers of neurons signaled different distances of the hand to the object for a given viewpoint. Remarkably, the large majority of F5c AOENs also responded robustly to an ellipse moving along the same trajectory as the hand in the action video, even in the absence of a graspable object, indicating that meaningful actions are not necessary for most AOENs. These results suggest that most F5c AOENs respond to remarkably elementary visual stimuli.

We observed previously unreported highly phasic responses during action observation that correlated well with the responses to static frames of the videos. Such precisely time-locked responses during a specific phase of the action can only be measured with repeated presentations of the same stimulus and exact timing of stimulus onset (which we achieved by means of a photo cell) but may have been overlooked in studies using naturalistic action observation where an actor performs the grasping action. Even more surprisingly, the overwhelming majority of AOENs in F5c—selected based on a criterion that has been used in numerous previous studies—also responded to the simple motion of a shape in the absence of the object in which no meaningful action, causality, or intentionality could be discerned, which challenges the notion that AOENs provide an abstract representation of an action or its intention. Our data do not allow determining whether an even more reduced stimulus would activate AOENs, but the videos of the ellipse moving on a scrambled background were already heavily reduced since they only contained motion of a shape towards and away from the center of the display. Furthermore, our main finding is that meaningful actions are not necessary for most AOENs, and this conclusion does not depend on testing all other potential visual stimuli.

The wider significance of our findings lies undoubtedly in their implications for the potential role of F5c AOENs in visuomotor control. To understand the functional role of a population of neurons, numerous studies in the visual system have determined the minimal visual stimuli that drive the responses ([23–25] for inferotemporal cortex [26]; for V4 [27]; for AIP [28]; for F5a [29]; for area MST). Our population of F5c AOENs frequently did not require meaningful actions but fired maximally when the hand was at a specific location in the video (depending on the viewpoint). The peak activity around hand–object interaction in the majority of AOENs could have been due to the processing of hand–object interactions, or to other factors such as signaling the stop of the hand or simply the position of the hand in central vision, but the relative lack of selectivity for Human Grasp compared to Human Touch videos (in which the interaction with the object was different but the location of the hand identical) suggests that AOENs do not encode specific hand–object interactions. During prehension, the grip aperture follows a highly standardized pattern, in which the aperture first increases and then decreases to match the size of the to-be-grasped object [30–32]. Therefore, it is important for the motor system to receive continuous visual feedback about the location of the hand relative to the object [30,33,34]. AOENs in F5c provide this information with high accuracy. As a result, we predict that the output of the F5c AOENs should be primarily directed towards other motor areas such as F4 (see [35]). The fact that AOENs in F5c are highly active during grasping in the dark may appear to contradict this hypothesis but might be reconciled with our findings if we consider the possibility that AOE responses can be multimodal (e.g., visual and proprioceptive information; see [36] for visual and auditory responses). Thus, AOENs in F5c that are also active in the dark may signal the position of the hand with respect to the object based on both visual and proprioceptive information.

To ensure that our findings would be as relevant as possible for the field, we selected recording sites based on a simple, reproducible, and widely used criterion—responsiveness during action execution and action observation—similar to the approach in most fMRI studies [16] (but restricting the analysis to AOENS that were also active in the dark yielded similar conclusions). Furthermore, we recorded both SUA and MUA and did not exclude any recording site based on other criteria such as responsiveness to the ellipse videos, or potential EMG activity, since most previous studies also did not exclude neurons based on these criteria [2]. Overall, we are convinced that our data set—with 213 single neurons and 289 MUA sites recorded in 4 animals—is representative for AOE neurons in the F5c sector of PMv.

Regardless of the inclusion criteria used, the fact that we recorded large numbers of AOENs (up to 23 in a single session) simultaneously made the potential contribution of aspecific factors such as reward expectation, attention, or muscle activity to the phasic responses during action observation unlikely. Simultaneously recorded AOENs fired maximally at different moments in time during the action videos, whereas a single aspecific factor (e.g., the allocation of attention) would affect neuronal activity at the same moment in time (e.g., around reward delivery, which occurred more than 500 ms after the end of the action video).

The use of the 96-channel chronically implanted multielectrode arrays in F5c posed both opportunities and limitations. Because the electrodes are not movable, we could not search for responsive neurons, making our approach less biased compared to previous single-electrode studies. Moreover, the 4 by 4 mm array covers a large part of F5c, and the 5 slightly different implantation locations in the 4 animals ensured that taken together, we must have covered most of the F5c subsector. A limitation of our approach was that—as in all single-cell studies—our recordings were inevitably biased towards neurons with large action potentials. However, our multiunit data contained all the spikes in the signal and were very similar to the single-unit results, which makes it highly unlikely that this potential bias would have a major impact on the results. Note also that the presentation of action videos allowed us to investigate epoch-specific phasic activity, but informal clinical testing of AOEN sites confirmed their responsiveness during grasping observation with an actual actor.

Our results do not allow to draw definitive conclusions about the underlying mechanisms of the observed AOE responses to simple translation of an ellipse, but a number of alternative explanations appear to be less likely. For example, we may have recorded from a very specific subpopulation of AOENs, possibly located in a single cortical layer, so that our results may not apply to the entire population. This possibility appears highly unlikely given that we acquired similar data with 5 implantations in 4 animals. The 96-electrode arrays we used covered a cortical area of 4 by 4 mm at different anteroposterior locations in F5c, which also makes it unlikely that we recorded from a specific subpopulation of F5c neurons. A final possibility is that the moving ellipse may have become associated with the hand in the action video through learning since action videos and control videos were presented in the same sessions in an interleaved way. We did not systematically record before the presentation of the ellipse videos, which makes it difficult to entirely rule out this learning explanation. However, 1 monkey (Monkey 4) was not exposed to any ellipse video before the recordings started, and yet we found ellipse neurons even in the first recording session. Moreover, if the translation of the ellipse somehow became associated with the grasping action, we would expect similar responses to the 2 viewpoints of the ellipse videos (Viewpoint 1 and Viewpoint 2), which was not the case. This type of learning mechanism would also not explain why different AOENs were tuned to different epochs of the action or ellipse video. It should also be noted that learning in a purely passive context (i.e., exposure) and without explicit reward causes relatively small effects on neuronal responses [37].

Caggiano and colleagues [18] have reported that AOENs in PMv are tuned for visual features related to the perception of causality, but in our ellipse video, there was no interaction with the object, and in the ellipse on scrambled background video, the to-be-grasped object was absent. However, our study differed from [18] with respect to the selection of the neurons. Caggiano and colleagues excluded all neurons that were not selective for the direction of movement of the sphere (towards versus away from the target object), while such selectivity was extremely rare in our population of AOENs. We adopted a very straightforward criterion to identify AOE activity (significant responses during grasping execution and grasping observation), which is very similar to the definition of mirror neuron activity in previous studies [38], but we did not require selectivity for approach versus recede.

The fact that most neurons responded to a simple moving stimulus in the absence of any interaction with the object suggests at the very least that these neurons do not require meaningful actions and therefore are not likely to provide an abstract representation of an observed action. On the other hand, our findings do not rule out the possibility that a minority of AOENs in F5c encodes meaningful actions. However, even non-ellipse AOENs generally responded to some degree to the moving ellipse and showed highly phasic discharges during specific epochs in the action videos, as if they were signaling particular phases of the action rather than the entire action. Furthermore, we cannot exclude the possibility that other simple visual stimuli (moving bars, spheres, or other shapes) could activate non-ellipse AOENs more strongly. It is also remarkable that the results we obtained in F5c were virtually identical to the ones of a previous study in AIP [12], which sends visual information to PMv and may be a potential input area to the AOE network [39,40]. Similar to F5c AOENs, 76% of AIP neurons responding during grasping observation and execution were also active during observation of an ellipse, even when moving on a scrambled background, and most of these AIP neurons responded maximally when the ellipse appeared close to the to-be-grasped object. A similar correspondence in neuronal selectivity between anatomically connected [41] parietal and F5 subsectors has been reported for 3D [28,42,43] and 2D [27,44] shape. Future studies will have to determine to what extent the different subsectors of F5 respond differently during action observation at the single-neuron level [9,45].

Since most AOENs fire to very simple moving stimuli in a specific epoch, our results suggest a role for AOENs in F5c in providing continuous visual feedback for online visuomotor control during object grasping. Although demonstrating a role in visually guided grasping does not preclude a role in action recognition [46,47], our results may represent the first step towards an entirely new view on the AOE system. The extended action-–observation neuronal network may have a primary role in monitoring one’s own actions (e.g., how far is the hand from the object) rather than in interpreting the actions of others. Future studies will have to determine to what extent simple movements are sufficient for other neurons of the AOEN network.

## Materials and methods

### Method details

#### Ethics statement

All surgical and experimental procedures were approved by the ethical committee on animal experiments of the KU Leuven (project number P047/2021) and performed according to the National Institute of Health’s Guide for the Care and Use of Laboratory Animals and the EU Directive 2010/63/EU.

#### Surgery and recording procedures

Four male rhesus monkey (*Macaca mulatta*, 8 kg) were implanted with a titanium head post that was fixed to the skull with dental acrylic and titanium screws. After training in a passive fixation task and a grasping task, a 96-channel microelectrode Utah array with 1.5 mm electrode length and an electrode spacing of 400 μm (4 × 4 mm; Blackrock Neurotech, UT, USA) was inserted during general anesthesia and guided by stereotactic coordinates and anatomical landmarks. We inserted the arrays using a pneumatic inserter (Blackrock Neurotech) with a pressure of 1.034 bar and an implantation depth of 1 mm. During all surgical procedures, the monkey was kept under propofol anesthesia (10 mg/kg/h) and strict aseptic conditions. Postoperative anatomical scans (Siemens 3T scanner, 0.6 mm resolution) verified the position of the Utah array in ventral premotor area F5c (Fig 1A, top left panel), contralateral to the monkey’s working hand. The remaining 3 panels of Fig 1A show the exact location of the 5 implantations in the 4 monkeys. The posterior edge of the 4 × 4 mm arrays was located 0 to 5 mm anterior to a vertical line extending down from the spur of the arcuate sulcus. Thus, our recording sites covered a substantial part of the inferior frontal convexity. Due to an implant failure in the second monkey, another Utah array was implanted in area F5c in the other hemisphere (“Monkey 2 Left” in Fig 1A).

During a recording session, data were collected using a 96-channel digital headstage (Cereplex M) connected to a digital neural processor and sent to a Cerebus data acquisition system (Blackrock Neurotech, UT, USA). Single- and multiunit signals were high pass filtered (750 Hz) and sampled at 30 kHz. The threshold to detect multiunit activity was set to 95% of the average noise level. The data were subsequently sorted offline with a refractory period of 1 ms to isolate single units, using the Offline Spike sorter software (Plexon, Dallas, TX, USA). Overall, we had a good yield over the implanted array in each monkey with approximately 60 channels with detectable single units in every session.

#### Experimental setup

The monkey was trained to sit upright in a primate chair with his head fixed during all experimental procedures. During the recording of neuronal activity, the monkey had to perform 2 different tasks sequentially in blocks (typically 20 trials per condition): a grasping task and a passive fixation task.

For the grasping task, a custom-built object containing 3 identical small spheres was placed in front of the monkey at a 28-cm viewing distance [48]. The spheres (2.5 cm diameter) were attached to a disk (15 cm diameter) with springs, allowing the monkey to pull the spheres. Each sphere contained a blue LED that could be turned on and off individually and was positioned at an angle of 120 degree relative to the other 2 spheres. In the center of the disk, a green LED served as the fixation point, and the dimming of this green LED was the go-signal for grasping. During the grasping task (Fig 1B), the monkey had to grasp one of the 3 identical spheres (indicated with the blue LED) in a pseudorandom order. The position of the hand was monitored using infrared laser beams, which were interrupted when the hand was positioned on the resting position. An infrared-based camera system (Eyelink 1000; SR Research, Ontario, Canada) monitored the eye movements to ensure fixation on the object during the duration of each trial.

For the passive fixation task, a display (17.3 inch) was placed in front of the monkey at the same viewing distance as the object in the grasping task. The monkey had to maintain fixation on a red dot in the center of the screen during the presentation of different videos. Eye movements were monitored to ensure fixation inside an approximately 2 degree fixation window using the same infrared-based camera system as in the VGG task. A photodiode attached to the lower right corner of the screen registered the onset of each video by the detection of a bright square (not visible to the monkey) that appeared simultaneously with the onset of the video. Photodiode pulses were sampled at 30 kHz on the Cerebus data acquisition system to allow synchronization with the neural data.

#### Visually guided and memory-guided grasping task (VGG and MGG)

In every recording session, the monkey had to perform a delayed visually guided reach-to-grasp task (Fig 1B). To start a trial, the monkey had to place its hand on a resting position in complete darkness. After 500 ms of fixation on a green LED in the center of the disk, an external light illuminated the object. At the same time, a blue LED appeared on one of the 3 spheres, indicating the sphere to-be-grasped. After a variable time (700 to 1,000 ms), the green LED dimmed (i.e., the go cue), instructing the monkey to release the resting position, grasp the object with the illuminated blue LED, and pull it to obtain a juice reward. The complete movement, from releasing the resting position to pulling the object, could maximally last 1,000 ms to ensure the shortest and most efficient reach trajectory. During the grasping task, the opposite hand was gently restrained to avoid movement. In the memory-guided version of the grasping task (MGG), all events were identical to the VGG task, with the exception of the light above the object and the blue LED on the target, which both went off after 300 ms, so that the animal had to grasp and pull the object in the dark after a delay of 700 to 1,000 ms. To avoid any influence of the reward on the activity around the pull of the object, reward was administered at least 40 ms after the detection of the pull of the object.

#### Action observation task

To initiate a trial, the monkey had to fixate on a small red dot that appeared in the center of the screen. After 300 ms of passive fixation, a video started. The monkey had to maintain its gaze on the fixation point during the presentation of the stimulus (15.5 × 10.3 visual degrees). In total, 12 conditions (1 video per condition) were shown in pseudorandom order during the task (Fig 1C; for example videos, see S1–S4 Videos). In brief, the stimulus set included 6 videos filmed from the point of view (Viewpoint 1) of the monkey and 6 videos filmed from the side (Viewpoint 2). Both viewpoints included a monkey and a human performing the VGG task (“Monkey grasp” and “Human grasp,” respectively), a human performing the same task without pulling the sphere (“Human touch”) and a video without any movement and no human or monkey hand visible (“Static”). After pulling the sphere, the hand moved back to the starting position. Additionally, 4 videos were shown in which an ellipse (a scrambled version of the monkey hand, major axis ± 95 mm, minor axis ± 43 mm, as in [12]) moved towards the object with the same kinetic parameters as the hand in the action videos. Since the period before movement onset in the ellipse videos differed from the one in the action videos, the total length of the ellipse videos was slightly different compared to the action videos. The background of the video was either the natural (“Ellipse”) or a scrambled version of the natural background (“SCR background”). We also presented videos of a static frame of the Human Grasp action video in which the hand was halfway towards the object and in which the hand was interacting with the object. All videos were made using the object with the 3 spheres from the grasping task and lasted between 2.6 and 3.5 s. Both arms of the monkey were restrained during the action observation task to prevent movement.

### Quantification and statistical analysis

All data were analyzed using custom written Matlab scripts (the MathWorks R2019b, MA, USA, Table 1). For each trial, we calculated the net spike rate in 50 ms bins by subtracting the baseline activity (average spike rate of 200 ms interval before object onset or before onset of the action video) from the spike rate after stimulus start (either object or action video). We analyzed 3 recording sessions for each implantation. Because the recording signal was unstable in the first weeks after implantation, we considered all spikes recorded on different days as different units. However, we verified that the results were essentially the same when analyzing a single recording session for each of the 3 implantations. All analyses were calculated on SUA and MUA independently and averaged across the 3 spheres that had to be grasped. Task-related neurons were significantly positively modulated (at least 5 spikes/s, and 3 SEs above baseline activity for at least 150 ms) during the VGG task in any of 3 epochs of the task (Go cue, Lift of the hand, and Pull). AOENs were defined as task-related (VGG) and significantly positively modulated (at least 5 spikes/s, and 3 SEs above baseline activity for at least 200 ms) during passive viewing of any of the action videos. Likewise, neurons were considered significantly negatively modulated during a task when the minimal spike rate was no more than 5 spikes/s and the average activity was at least 3 SEs below the baseline activity for at least 200 ms. The average net spike rate was calculated for 15 to 35 repetitions per condition for each task.

**Table 1:** Key resources table.

RESOURCE	SOURCE	IDENTIFIER
Software		
EEGlab toolbox		https://sccn.ucsd.edu/eeglab/index.php
Matlab R2019b	Mathworks	https://www.mathworks.com

Software used for analysis of the EMG and neural recordings.

To assess the selectivity of our AOEN sample, we calculated the average responses to each action video as the average spike rate in a 200-ms interval around the maximum and then calculated a 2-way ANOVA on these average responses with factors *viewpoint* (Viewpoint 1 and Viewpoint 2) and *action type* (Human touch, Human Grasp, and Monkey Grasp). This way, we tested for viewpoint selectivity and congruence of the action during execution and observation. Additionally, we calculated the d’ selectivity index for each neuron to quantify viewpoint selectivity:

$$d^{\prime }=\frac{mean(VP1)-mean(VP2)}{\sqrt{(var(VP1)+var(VP2))/2}}$$

with VP1 = viewpoint 1 and VP2 = viewpoint 2. We determined the preferred video as the one eliciting the highest firing rate for each SUA or MUA site. To capture the phasic nature of the responses during action observation, we used the matlab function “findpeaks” detecting peaks in the normalized (by dividing by the maximum) net firing rate to each video with a minimal prominence (i.e., the decline in spike rate on either side of the peak) of 0.8, discarding sites that had more than 3 peaks due to noisy responses. We analyzed the peaks that were identified by the Matlab function regardless of the time epoch to account for the variable length of the videos. To characterize the degree of tuning during passive viewing of the action video, we calculated the FWHM of the spike rate around the peak response to the preferred action video. For all AOENs, we then plotted the x and y position of the hand with respect to the object and the Euclidean distance (in pixels) to the object 50 ms before the peak response occurred (to account for the latency of the neuronal response). A Kruskal–Wallis 1-way ANOVA was used to test whether the neurons showed a significant preference for one of the 3 movement intervals: approaching the object, interacting with the object, and receding from the object. Additionally, to test whether static frames of the action video could account for the observed responses, we plotted the peak firing rate during the preferred action video against the peak firing rate during the static frame videos (in which the hand was either touching the object or halfway its trajectory towards the object). We then calculated the Pearson correlation coefficient between the 2 peak firing rates (action video and static frame video) for each static frame video. This control experiment was performed in a subset of the recorded F5c sample.

Because almost no AOEN responded during the entire video but rather during specific epochs, we compared the maximal spike rate during the preferred action video to the maximal spike rate during the corresponding ellipse video with the normal background. Note that our analysis ignored the exact timing of the maximal firing rate because the action videos and the ellipse videos differed in length. Ellipse neurons were defined as AOENs with a maximal spiking response to the ellipse video that was at least 50% of the maximal spike rate during viewing of the preferred action video, analogous to [12]. Furthermore, to assess whether the F5c ellipse neurons were selective for the direction or the orientation of the movement, we used a Mann–Whitney U test to compare the average activity of each neuron during the approaching and the receding phase of the ellipse when it moved on the scrambled background. Since our action execution task only included object grasping and in line with [2], we defined strictly congruent AOENs as neurons that showed a significant preference for Human grasp over Human touch (based on a 2-way ANOVA with factors *perspective* and *action type*, main effect of *action type p* < 0.05). Broadly congruent AOENs were defined as neurons in which the main effect of *action type* was not significant. All post hoc tests were calculated with the Tukey’s honestly significant difference procedure.

Suppression AOENs were defined as neurons that were significantly positively modulated during the action execution task and significantly negatively modulated during the action observation task, as in [8]. To assess whether suppression AOENs also responded to the ellipse control video, we calculated the Pearson correlation coefficient between the average spike rate in a 200-ms interval around the most inhibitory activity during the preferred action video and the average spike rate in the corresponding interval in the ellipse video. In contrast to excitatory AOENs, suppression AOENs did not exhibit a phasic response to the videos. Therefore, we calculated the average spike rate in an interval instead of using the spike rate in 1 bin. For each AOEN, we defined the preferred action video as the action video with the lowest net spike rate during the movement of the hand.

To investigate whether muscle activity contributed to the neural responses observed during the action observation task, we measured the EMG activity of the thumb and bicep muscle of the hand used in the VGG task during passive fixation of the action videos. The EMG signal was recorded in Monkey 3 using dry self-adhesive electrodes. The ground electrode was placed next to the recording electrode on the bicep muscle. Data were obtained with a multichannel amplifier (EMG100C, BIOPAC Systems, CA, US) and sampled at 10,000 Hz with a gain of 5,000 (Table 1). After applying a bandpass filter between 2 and 30 Hz, the rectified EMG signal was aligned to the neural data. We then correlated the rectified EMG signal (in 50 ms bins) with the spiking activity of each AOEN in a 1,000-ms interval (500 ms around the peak response and 500 ms 1 s before the peak response).

## Supporting information

S1 Fig Inhibitory grasping activity in F5c and the corresponding action observation responses. (**A**) Top: color plots of the net spikes rate of each SUA that was negatively modulated during the VGG task. Bottom: average net spike rate (±SEM) of all negatively modulated neurons, aligned on the 4 events of the VGG task: Object Onset, Go cue, Lift of the hand, and Pull. (**B**) Maximal spiking activity during the preferred action video plotted against the maximal spiking activity during the corresponding ellipse video. The orange line represents the 50% criterion to define ellipse neurons. (**C**) Peak spiking activity during the ellipse video (perspective of the preferred action video) plotted against the peak firing rate during the corresponding scrambled background video for ellipse AOENs. Dashed lines represent the equality lines.
(DOCX)

S2 Fig Neural properties of F5c AOENs concerning congruence of action, actor, and viewpoint. (**A**) Correlation of the maximal spiking activity during observation of the Human Grasp video and the Human Touch video, perspective corresponding to the perspective of the preferred action video for each site. (**B**) Same as in (**A**) but comparing the Human Grasp video and the Monkey Grasp video. Dashed lines represent the equality lines. (**C**) Distribution of d’ selectivity index of all AOENs.
(DOCX)

S3 Fig Contribution of aspecific factors, such as muscle contractions, attention, or reward delivery. (**A**) Euclidean distance between the hand and the object in the preferred action video when the neuron discharged maximally, for each neuron in each session (S1-S3: Monkey 1, S4-S6: Monkey 2 Right, S7-S9: Monkey 2 Left, S10-S12: Monkey 3, S13-S15: Monkey 4. A distance of zero indicates interaction between the hand and the object. (**B**) Average neural signal during the preferred action video of an example AOEN and simultaneously recorded EMG signal of the thumb and bicep muscles. Data are aligned on video onset.
(DOCX)

S4 Fig Suppression AOENs respond to the movement of an abstract shape. (**A**) Net spike rate (±SEM) for 2 example neurons during the observation of the action video with the most inhibitory response (blue) and the corresponding ellipse video (ocher). The vertical black line represents the moment of object interaction in the action video. (**B**) Average spike rate in a 200-ms interval around the most inhibitory spike rate during the preferred action video plotted against the average spike rate in the corresponding interval during the corresponding ellipse video. The dashed line represents the equality line.
(DOCX)

S5 Fig MUA responses in F5c. (**A**) Normalized average net spike rate (±SEM) of the positively modulated MUA sites of each monkey, aligned on the 4 events of the VGG task: Object Onset, Go cue, Lift of the hand, and Pull. (**B**) Left: average peak response (±SEM) of 221 MUA sites with AOE activity plotted in a 500-ms interval around the peak. Right: position of the hand relative to the object in the preferred action video at maximal spiking activity. Colors indicate the phase of the movement: green (Approach), blue (Object interaction), and ocher (Recede). Histogram in the inset shows the Euclidean distances between the hand and the object at maximal spiking activity. (**C**) Maximal spiking activity during the preferred action video plotted against the maximal spiking activity during the corresponding ellipse video. The orange line represents the 50% criterion to define MUA sites with ellipse activity. (**D**) Peak spiking activity during the ellipse video (perspective of the preferred action video) plotted against the peak firing rate during the corresponding scrambled background video for MUA sites with ellipse activity. Dashed lines represent the equality lines.
(DOCX)

S1 Video Human Grasp video of viewpoint 2. (AVI)

S2 Video Monkey Grasp video of viewpoint 2. (AVI)

S3 Video Ellipse video on the normal background of viewpoint 2. (AVI)

S4 Video Ellipse video on the scrambled background of viewpoint 2. (AVI)

## Abbreviations

AOEN: action observation/execution neuron
EMG: electromyographic
fMRI: functional magnetic resonance imaging
FWHM: full width at half maximum
SE: standard error
