# Supplementary material for: Simple visual stimuli are sufficient to drive responses in action observation and execution neurons in macaque ventral premotor cortex
Source: PLoS Biol. 2024 May 20;22(5):e3002358. doi: 10.1371/journal.pbio.3002358 (PMC11142659; doi:10.1371/journal.pbio.3002358)
Supplement: S1 Fig — (A) Top: color plots of the net spikes rate of each SUA that was negatively modulated during the VGG task. Bottom: average net spike rate (±SEM) of all negatively modulated neurons, aligned on the 4 events of the VGG task: Object Onset, Go cue, Lift of the hand, and Pull. (B) Maximal spiking activity during the preferred action video plotted against the maximal spiking activity during the corresponding ellipse video. The orange line represents the 50% criterion to define ellipse neurons. (C) Peak spiking activity during the ellipse video (perspective of the preferred action video) plotted against the peak firing rate during the corresponding scrambled background video for ellipse AOENs. Dashed lines represent the equality lines. (DOCX) [file pbio.3002358.s001.docx]

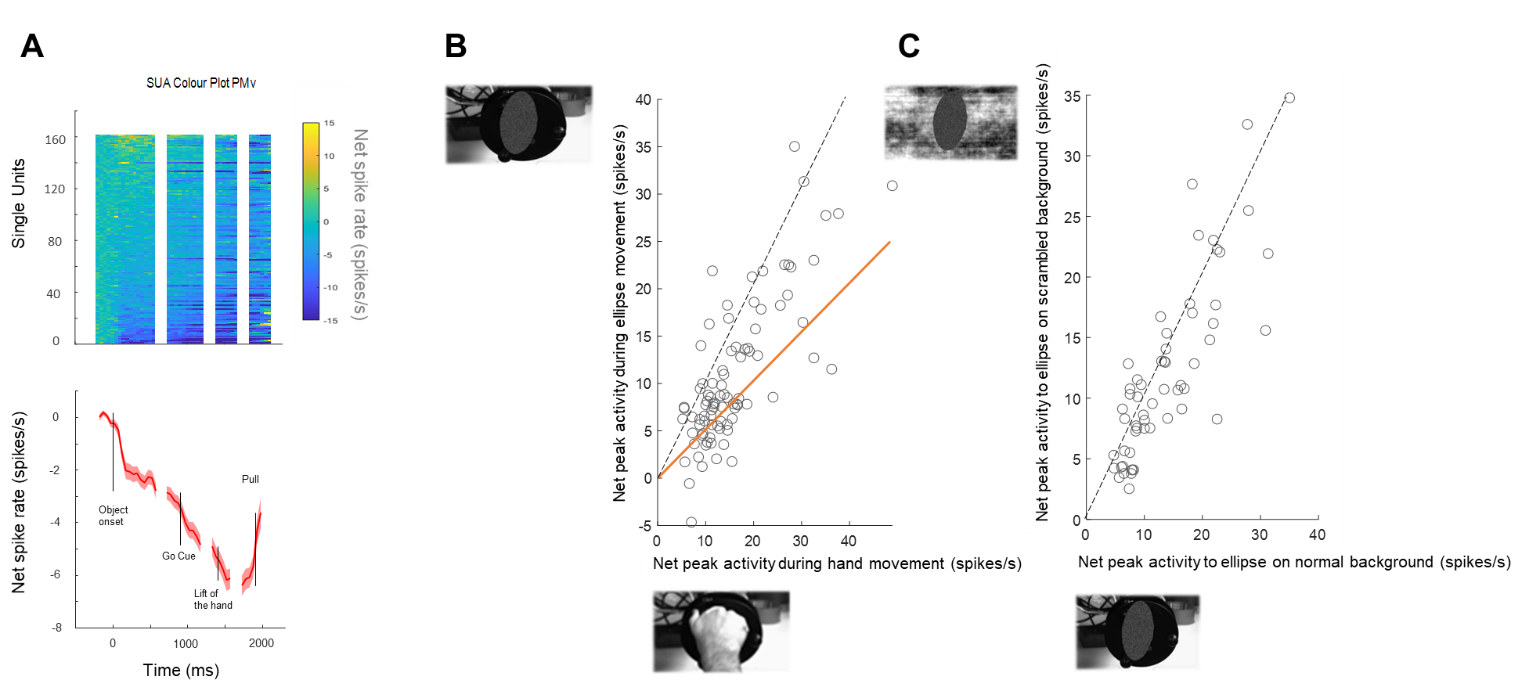


S1 fig: Inhibitory grasping activity in F5c and the corresponding action observation responses. (A) Top: color plots of the net spikes rate of each SUA that was negatively modulated during the VGG task. Bottom: Average Net spike rate (± SEM) of all negatively modulated neurons, aligned on the four events of the VGG task: Object Onset, Go cue, Lift of the hand, and Pull. (B) Maximal spiking activity during the preferred action video plotted against the maximal spiking activity during the corresponding ellipse video. The orange line represents the 50% criterion to define ellipse neurons. (C) Peak spiking activity during the ellipse video (perspective of the preferred action video) plotted against the peak firing rate during the corresponding scrambled background video for ellipse AOENs. Dashed lines represent the equality lines.
