# Supplementary material for: Simple visual stimuli are sufficient to drive responses in action observation and execution neurons in macaque ventral premotor cortex
Source: PLoS Biol. 2024 May 20;22(5):e3002358. doi: 10.1371/journal.pbio.3002358 (PMC11142659; doi:10.1371/journal.pbio.3002358)
Supplement: S2 Fig — (A) Correlation of the maximal spiking activity during observation of the Human Grasp video and the Human Touch video, perspective corresponding to the perspective of the preferred action video for each site. (B) Same as in (A) but comparing the Human Grasp video and the Monkey Grasp video. Dashed lines represent the equality lines. (C) Distribution of d’ selectivity index of all AOENs. (DOCX) [file pbio.3002358.s002.docx]

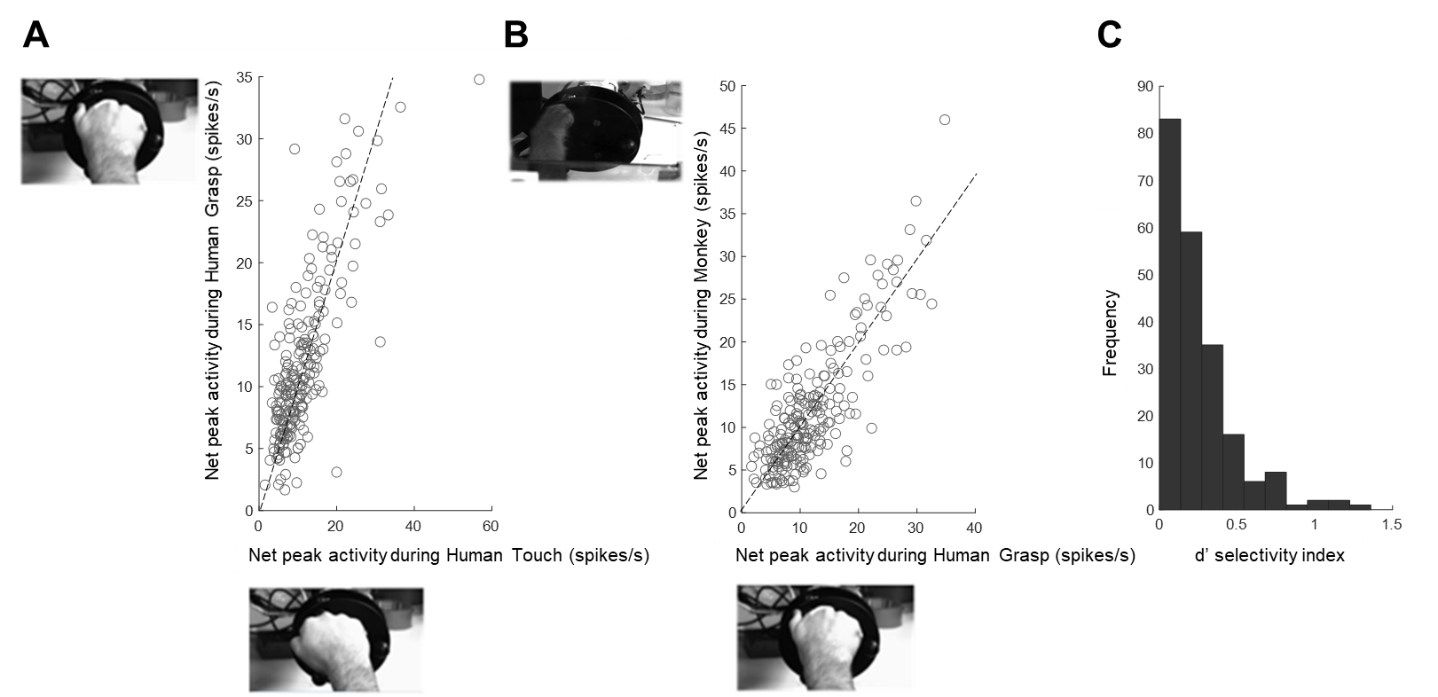


S2 fig: Neural properties of F5c AOENs concerning congruence of action, actor, and viewpoint. (A) Correlation of the maximal spiking activity during observation of the Human Grasp video and the Human Touch video, perspective corresponding to the perspective of the preferred action video for each site. (B) Same as in (A) but comparing the Human Grasp video and the Monkey Grasp video. Dashed lines represent the equality lines. (C) Distribution of d’ selectivity index of all AOENs.
