# Supplementary material for: Simple visual stimuli are sufficient to drive responses in action observation and execution neurons in macaque ventral premotor cortex
Source: PLoS Biol. 2024 May 20;22(5):e3002358. doi: 10.1371/journal.pbio.3002358 (PMC11142659; doi:10.1371/journal.pbio.3002358)
Supplement: S3 Fig — (A) Euclidean distance between the hand and the object in the preferred action video when the neuron discharged maximally, for each neuron in each session (S1-S3: Monkey 1, S4-S6: Monkey 2 Right, S7-S9: Monkey 2 Left, S10-S12: Monkey 3, S13-S15: Monkey 4. A distance of zero indicates interaction between the hand and the object. (B) Average neural signal during the preferred action video of an example AOEN and simultaneously recorded EMG signal of the thumb and bicep muscles. Data are aligned on video onset. (DOCX) [file pbio.3002358.s003.docx]

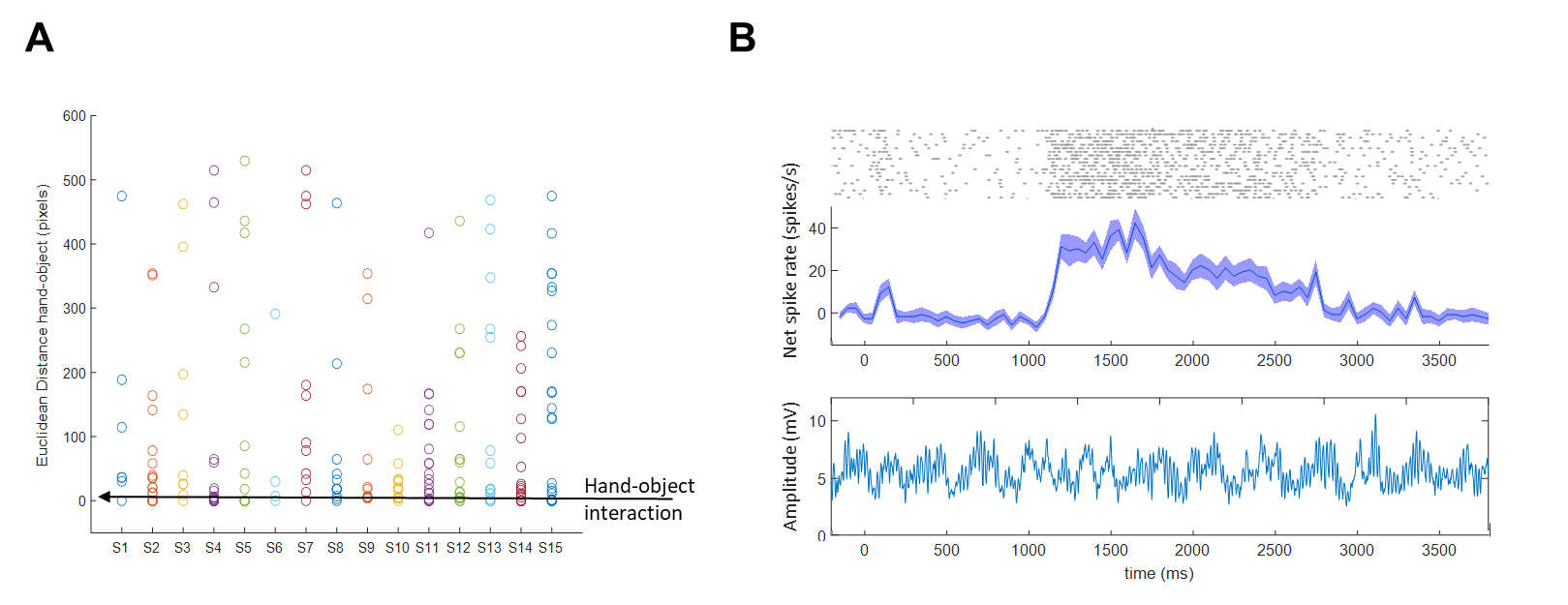


S3 fig: Contribution of aspecific factors, such as muscle contractions, attention or reward delivery. (A) Euclidean distance between the hand and the object in the preferred action video when the neuron discharged maximally, for each neuron in each session (S1-S3: Monkey 1, S4-S6: Monkey 2 Right, S7-S9: Monkey 2 Left, S10-12: Monkey 3, S13-15: Monkey 4. A distance of zero indicates interaction between the hand and the object. (B) Average neural signal during the preferred action video of an example AOEN and simultaneously recorded EMG signal of the thumb and bicep muscles. Data is aligned on video onset.
