# Supplementary material for: Simple visual stimuli are sufficient to drive responses in action observation and execution neurons in macaque ventral premotor cortex
Source: PLoS Biol. 2024 May 20;22(5):e3002358. doi: 10.1371/journal.pbio.3002358 (PMC11142659; doi:10.1371/journal.pbio.3002358)
Supplement: S4 Fig — (A) Net spike rate (±SEM) for 2 example neurons during the observation of the action video with the most inhibitory response (blue) and the corresponding ellipse video (ocher). The vertical black line represents the moment of object interaction in the action video. (B) Average spike rate in a 200-ms interval around the most inhibitory spike rate during the preferred action video plotted against the average spike rate in the corresponding interval during the corresponding ellipse video. The dashed line represents the equality line. (DOCX) [file pbio.3002358.s004.docx]

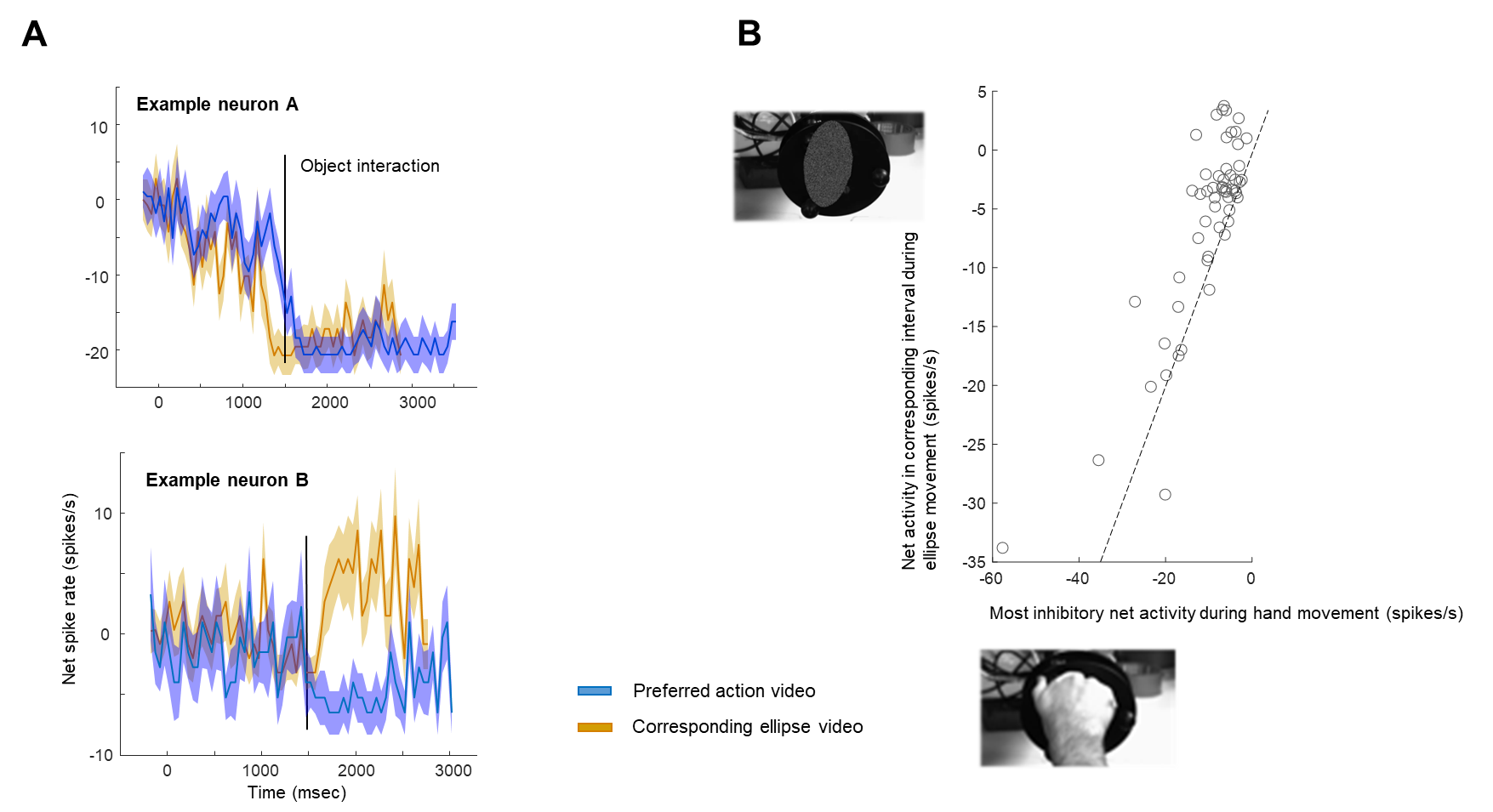


S4 fig: Suppression AOENs respond to the movement of an abstract shape. (A) Net spike rate (± SEM) for two example neurons during the observation of the action video with the most inhibitory response (blue) and the corresponding ellipse video (ocher). The vertical black line represents the moment of object interaction in the action video. (B) Average spike rate in a 200ms interval around the most inhibitory spike rate during the preferred action video plotted against the average spike rate in the corresponding interval during the corresponding ellipse video. The dashed line represents the equality line.
