# Supplementary material for: Simple visual stimuli are sufficient to drive responses in action observation and execution neurons in macaque ventral premotor cortex
Source: PLoS Biol. 2024 May 20;22(5):e3002358. doi: 10.1371/journal.pbio.3002358 (PMC11142659; doi:10.1371/journal.pbio.3002358)
Supplement: S5 Fig — (A) Normalized average net spike rate (±SEM) of the positively modulated MUA sites of each monkey, aligned on the 4 events of the VGG task: Object Onset, Go cue, Lift of the hand, and Pull. (B) Left: average peak response (±SEM) of 221 MUA sites with AOE activity plotted in a 500-ms interval around the peak. Right: position of the hand relative to the object in the preferred action video at maximal spiking activity. Colors indicate the phase of the movement: green (Approach), blue (Object interaction), and ocher (Recede). Histogram in the inset shows the Euclidean distances between the hand and the object at maximal spiking activity. (C) Maximal spiking activity during the preferred action video plotted against the maximal spiking activity during the corresponding ellipse video. The orange line represents the 50% criterion to define MUA sites with ellipse activity. (D) Peak spiking activity during the ellipse video (perspective of the preferred action video) plotted against the peak firing rate during the corresponding scrambled background video for MUA sites with ellipse activity. Dashed lines represent the equality lines. (DOCX) [file pbio.3002358.s005.docx]

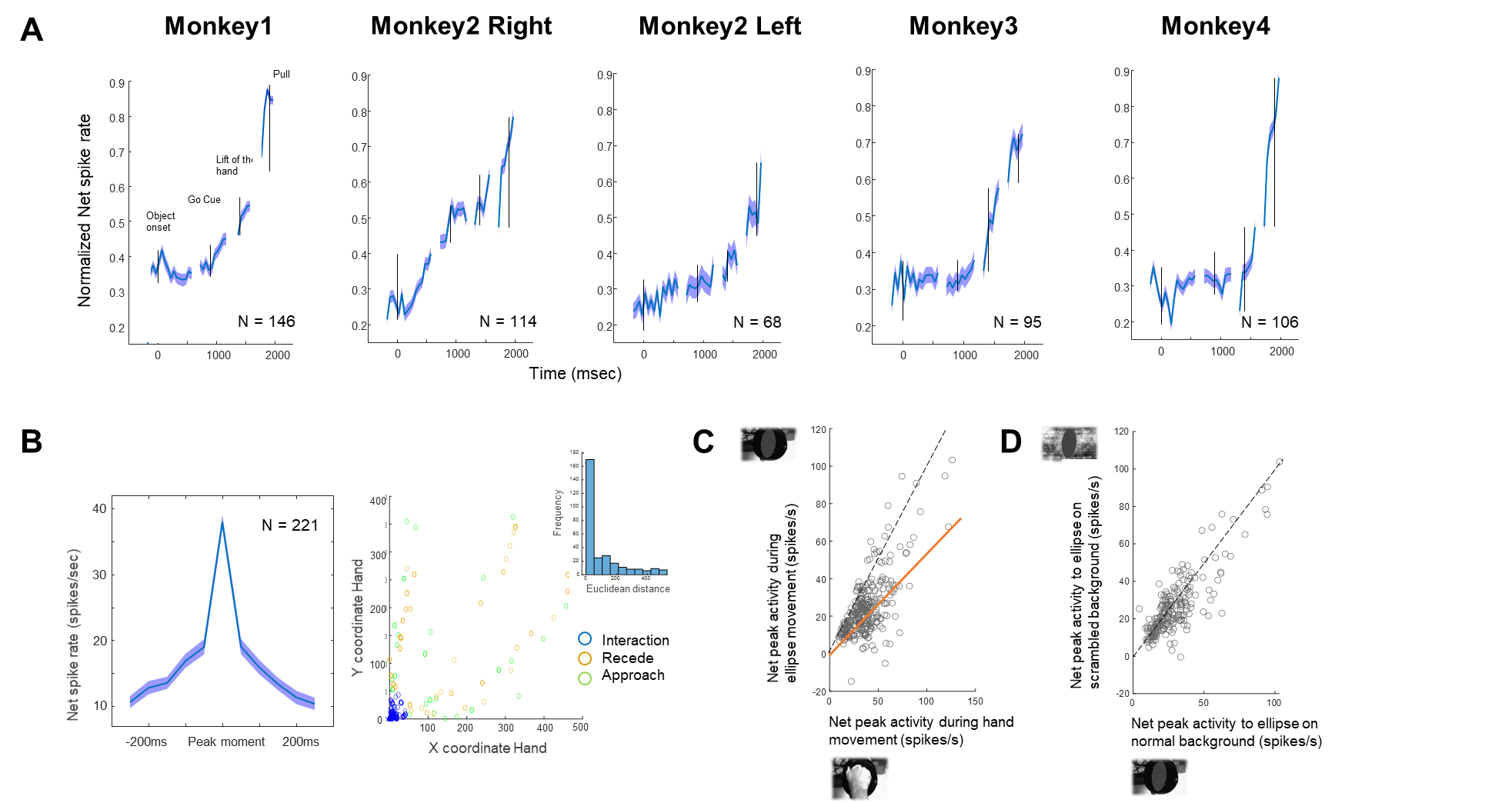


S5 fig: MUA responses in F5c. (A) Normalized average Net spike rate (± SEM) of the positively modulated MUA sites of each monkey, aligned on the four events of the VGG task: Object Onset, Go cue, Lift of the hand, and Pull. (B) Left: Average peak response (± SEM) of 221 MUA sites with AOE activity plotted in a 500ms interval around the peak. Right: Position of the hand relative to the object in the preferred action video at maximal spiking activity. Colors indicate the phase of the movement: green (Approach), blue (Object interaction), and ocher (Recede). Histogram in the inset shows the Euclidean distances between the hand and the object at maximal spiking activity. (C) Maximal spiking activity during the preferred action video plotted against the maximal spiking activity during the corresponding ellipse video. The orange line represents the 50% criterion to define MUA sites with ellipse activity. (D) Peak spiking activity during the ellipse video (perspective of the preferred action video) plotted against the peak firing rate during the corresponding scrambled background video for MUA sites with ellipse activity. Dashed lines represent the equality lines.
